# Non-Human Primate Models of Dengue Virus Infection: A Comparison of Viremia Levels and Antibody Responses during Primary and Secondary Infection among Old World and New World Monkeys

**DOI:** 10.3390/pathogens9040247

**Published:** 2020-03-27

**Authors:** Nor Azila Muhammad Azami, Tomohiko Takasaki, Ichiro Kurane, Meng Ling Moi

**Affiliations:** 1 UKM Medical Molecular Biology Institute, Universiti Kebangsaan Malaysia, Kuala Lumpur 56000, Malaysia; azila_azami@ukm.edu.my; 2 Kanagawa Prefectural Institute of Public Health, Kanagawa 253-0087, Japan; takasaki.jp58@pref.kanagawa.jp; 3 National Institute of Infectious Diseases, Tokyo 162-8640, Japan; kurane@nih.go.jp; 4 Institute of Tropical Medicine, Nagasaki University, Nagasaki 852-8523, Japan

**Keywords:** common marmoset, tamarin, cynomolgus macaque, dengue virus, secondary infection, non-human primate, animal model, vaccine study

## Abstract

Due to the global burden of dengue disease, a vaccine is urgently needed. One of the key points in vaccine development is the development of a robust and reliable animal model of dengue virus infection. Characteristics including the ability to sustain viral replication, demonstration of clinical signs, and immune response that resemble those of human dengue virus infection are vital in animal models. Preclinical studies in vaccine development usually include parameters such as safety evaluation, induction of viremia and antigenemia, immunogenicity, and vaccine effectiveness. Although mice have been used as a model, non-human primates have an advantage over mice because of their relative similarity to humans in their genetic composition and immune responses. This review compares the viremia kinetics and antibody responses of cynomolgus macaques (*Macaca fasicularis)*, common marmosets (*Callithrix jacchus)*, and tamarins (*Saguinus midas* and *Saguinus labitus*) and summarize the perspectives and the usefulness along with challenges in dengue vaccine development.

## 1. Introduction

Dengue is a major threat to global public health. Infection with any one of the four serotypes of dengue virus (DENV), DENV1-4, causes a wide variety of clinical illness, ranging from self-limited febrile illness, dengue with and without warning signs, to severe dengue and dengue-related death. According to the 2009 World Health Organization (WHO) guidelines, dengue without warning signs is defined as high-grade fever with nausea, vomiting, rash, and leucopenia [1]. The criteria for dengue with warning signs are abdominal pain, persistent vomiting, fluid accumulation, mucosal bleeding, lethargy, liver enlargement, and a raised hematocrit with a decreased platelet count [1]. Additionally, the hallmark of severe dengue is the presence of vascular leakage [2]. There are three phases of dengue illness: (1) a febrile phase, which lasts for 3 to 7 days; (2) a critical phase; and (3) a recovery phase [3]. While most patients recover after the febrile phase, a small proportion progress to severe dengue [3].

DENV belongs to the genus *Flavivirus* of the family *Flaviviridae*. Genus *Flaviviridae* encompasses antigenically closely related viruses that cause disease in humans, including the Japanese encephalitis virus (JEV), yellow fever virus (YFV), West Nile virus (WNV), and Zika virus (ZIKV). DENV is usually endemic in tropical and subtropical countries, including the South Pacific, East Mediterranean, Americas, and Southeast Asia. In recent years, autochthonous DENV outbreaks have been reported in temperate countries, including Japan, Croatia, and France [4,5,6]. DENV has infected 4 billion people worldwide with 390 million new cases of DENV infection reported annually [7,8,9,10]. The incidence of DENV infection has increased by up to 30-fold in the past 60 years [11].

As the global burden of DENV is continuously increasing, a dengue vaccine that is able to provide protection against all serotypes of DENV is required. A safe and efficacious dengue vaccine is important in the dengue control program. However, the development of a dengue vaccine has been hampered due to the lack of a reliable animal model. Vaccine trials include safety evaluation, induction of viremia and antigenemia, immunogenicity, and efficacy. Thus, an animal model that faithfully mirrors the immune response pattern of those of human DENV infection is able to sustain viral replication and exhibits age-related clinical signs would be the ideal model for vaccine trials because candidate vaccines are evaluated by defining the viremia kinetics and the antibody responses [12,13]. Mice have been used in vaccine trials, but low levels of DENV replication potential have led to inconclusive outcomes regarding the potency and immune response [14,15,16]. Non-human primates (NHPs) are preferred because of the high similarities in genetic and immune responses to those of humans. However, some NHP studies have induced low levels of viremia following virus inoculation and the trial subjects did not exhibit overt clinical signs [17,18,19]. In recent years, the common marmoset has shown promise as a potential animal model for DENV infection and candidate vaccine evaluation [13,20,21]. Here, we reviewed the viremia kinetics and antibody responses of cynomolgus macaques (*Macaca fasicularis)*, common marmosets (*Callithrix jacchus)*, and tamarins (*Saguinus midas* and *Saguinus labitus*) and assessed the utility of each NHP as a potential animal model in dengue vaccine trails.

## 2. Animal Models for Dengue Virus Infection

Animal models have been used in the research of DENV tropism, dengue pathogenesis, immune responses, therapeutics, and vaccine development and evaluation. An ideal animal model for DENV vaccine evaluation should demonstrate a high sensitivity to DENV and exhibit clinical signs and immune responses similar to those of human DENV infection. These characteristics are important for mimicking human DENV infection and for leading to a better understanding on the pathogenesis of DENV disease. The benefits and limitations of each of the animal models are summarized in Table 1.

Mice that have been used as models of DENV infection include immunocompetent mice, human tissue engrafted-severe combined immunodeficiency (SCID) mice, interferon α, β, γ deficient AG129, RAG-hu, and the NOD/SCID/ IL-2Rγ/human CD34 transplant mice [2,31,32,39,44,58,59]. Although the mouse model is the primary model used in therapeutics and vaccine efficacy studies, limited replication of DENV have compromised the outcome of these studies [14,15,16]. Initial studies adopted intracranial inoculation and this method induced neurological diseases and paralysis, clinical signs which are atypical signs of dengue fever and classical signs of dengue hemorrhagic fever [23,60,61]. As such, the end point of vaccine efficacy studies in immunocompetent mice are a reduced magnitude and the duration of viremia and neuropathological signs. The immunocompetent mouse model may be less sensitive to challenge by clinical DENV strains. Humanized mice enable studies on disease pathogenesis and several potential dengue biomarkers such as chemokines MCP-1, Th-2 and cytokines, Il-4, IL-10, and TNF-alpha have been observed in humanized mice following DENV inoculation [38,62]. However, low levels of antibody and the lack of other possible human targets cells, such as endothelial and hepatocytes, limits the utility of the model for vaccine evaluation study [38]. AG129 mice have, however, enabled an improved experimental model of dengue fever and dengue hemorrhagic fever study to be developed. The AG129 mouse develops severe dengue clinical signs, including vascular leakage, a hallmark of severe DENV infection [26,27]. Antibody response in AG129 mice also reflects those of the wild-type mice, although the T-cell response in the absence of interferon receptors needs to be considered in the interpretation of the results. While the use of immune-incompetent mice is a useful model, the ability to develop severe disease is also age-dependent. As vaccine evaluation requires observation of efficacy over a long time period, vaccine efficacy needs to be carefully interpreted with age-related clinical signs in mind.

The similarities in genetic, physiological, and immune responses between NHP and humans favor the use of the NHP as an animal model for DENV infection [42,43,44,49,63]. The presence of dengue antibodies in sera from wild NHP indicates their involvement in the sylvatic cycle [42,43]. The Old World monkey and New World monkey are the two group of NHP that have been used as DENV animal models. Rhesus macaque (*Macaca mulatta)* is the first NHP that was used in the studies of dengue etiology by the inoculation of defibrinated blood from dengue patient via intravenous and subcutaneous routes [64]. Rhesus macaque were widely used as an animal model for DENV, but they rarely developed the clinical manifestations observed in human dengue patients. Subcutaneous virus inoculation resulted in low levels of viremia in rhesus macaque, thus limiting the usage of this model in dengue vaccine studies [44,45]. In addition, NHP models, including pigtail macaque, rhesus macaque, and owl monkey have exhibited limited levels of viremia following inoculation with clinically isolated DENV strains [18,65]. Experiments using *Aotus* monkeys, squirrel monkeys, cotton-top tamarins, white face monkeys, black spider monkeys, Saimiri monkeys, marmosets (*Marikina geoffroyi*), howler monkeys, and red spider monkeys have demonstrated the susceptibility of New World monkeys to DENV, but none of these NHPs have exhibited overt clinical manifestations [42,47,48]. In recent years, rhesus macaques have been reported to show hemorrhagic signs following intravenous DENV inoculation [45]. DENV infection has been characterized with elevated levels of pro-inflammatory cytokines including IFN-γ, IL-6 IL-8 and TNFα. In addition, several potential dengue biomarkers, such as creatine phosphokinase (CK), TNF-alpha, IFM gamma, IL-8 and IL10, have been observed in NHPs following DENV inoculation [19,45,50,66]. Marmosets exhibited elevated levels of TGF-α and IFN-γ during the early phase of DENV infection and demonstrated altered serum biochemical, thrombocytopenia and leukopenia [13,20,21,50]. Marmosets with secondary DENV infection demonstrated infectious virus-immune complex and higher viremia titers, as detected by using Fc gamma receptor expressing cells. Further biomarker network analysis in the model revealed that IL-2/IL-6 were involved in the pathological axis and suggest that the later phase of infection involves IFN-γ, IL-4 and IL-5. The results indicate that elevated proinflamatory cytokines in NHP models may reflect some aspects of DENV pathogenesis in patients, although further manipulation is expected to improve the animal models. Clark et al. have comprehensively reviewed the utility of NHP as animal model of DENV from the perspective of pathology and immunopathology [44].

Due to the lack of reliable animal models to demonstrate the complexity of the immunologic process following DENV infection, dengue human infection models (DHIM) have been proposed for use in DENV vaccine efficacy trials [22,51,52,54,67]. The use of DHIM would enable vaccine development to be accelerated because they could provide insight into the correlates of protection and the pathogenesis of dengue [53,56,67,68]. The use of DHIM has also been hypothesized to be cost-effective as DHIM may provide an approach to select vaccine candidates based on their ability to reduce illness before embarking on large-scale clinical trials [55,56,67]. In addition, the use of DHIM would be useful for the studies on cellular immunity, the functional role of cytokines, and disease progression mechanism [53,67]. However, there are several safety issues regarding the use of DHIM, including the risk of severe dengue development in vaccinated subjects due to poor vaccine protection, differences in responses to DENV by different ethnic groups, and difficulty in obtaining human tissue biopsy samples for further pathological studies [22,68,69]. In addition, unlike human challenge models for influenza and malaria that have licensed specific therapy, there is no currently available licensed specific therapy for DENV [22,69]. Therefore, animal models remain important for preclinical testing of dengue vaccines.

## 3. Non-Human Primates

NHPs are divided into two groups: parvorder Platyrrhini (broad and flat-nosed) and parvorder Catarrhini (downward-nosed) [70]. The name of the New World monkey is derived from the fact that all Platyrrhinis live in South America and the name of the Old World monkeys refers to all African and Asian NHP [70]. The New World and Old World monkeys are estimated to have split between 33 to 77 million years ago [71]. All the Old World monkeys belong to the *Cercopithecidae* family, and the family is divided into two subfamilies of Colobines and Cercopithecines [70,71,72]. New World monkeys consist of five families: *Atelidae*, *Aotidae*, *Callithrichidae*, *Cebidae*, and *Pitheciidae* [73]. While Old World monkeys and New World monkeys are commonly used in infectious disease and biomedical research due to their close genetic proximity to humans, prosimians and great apes (chimpanzees) have been used less frequently. As the physiological similarities between humans and NHPs are greater than those of other animal models, NHPs are key to addressing research questions that cannot be addressed using other animal models.

Cynomolgus macaques (*Macaca fascicularis*), also known as long-tailed or crab-eating macaques, belong to subfamilies of *Cercopithecinae* and are the NHPs that are commonly used as DENV infection models in vaccine trials [70]. Cynomolgus macaques are native to the Southeast Asian mainland (Bangladesh, Myanmar, Thailand, Laos, Vietnam, Cambodia, and the Malaysian Peninsula), Sundaland (the islands of Borneo, Sumatra and Java, and the adjacent islands), and the Philippines [70,74]. Cynomolgus macaques reach sexual maturity at the age of 4 years in females and 6 years in males, and have a life-span ranging from 25 to 30 years [75,76]. As cynomolgus macaques and rhesus macaques are commonly used in biomedical research, a comparatively wide range of research tools are available for these NHPs.

The common marmoset (*Callithrix jacchus*) belongs to the *Callitrichidae* family. Marmosets are small in size, weighing about 350 to 400 grams, and are native to northeastern Brazil [77,78]. Marmosets reach sexual maturity between the age of 18 and 24 months and have a life expectancy of of 8 years [78,79]. Their compressed life-span, ability to breed well in captivity, small size, ease of handling, and lower cost of maintenance are attractive features of using marmosets in scientific research, and these traits allowed the introduction of variability into experimental procedures [21,77,80]. Marmosets have been used in the research of drug toxicology, aging, reproduction biology, behavioral research, neuroscience, autoimmune diseases, and infectious diseases [21,81,82,83,84].

Tamarins belong to the *Saguinus* genus of the *Callitrichidae* family. They weigh from 250 to 550 grams and are widely distributed across Central and South America, north and south of the Amazon, west of the Madeira River [85]. Tamarins (*Saguinus mystax*) have been used as experimental animals in GB virus B (GBV-B) studies, which have served as surrogates for hepatitis C virus studies [86]. The cotton-top tamarin (*Saguinus oedipus*) has been widely used as an experimental model in the research of inflammatory bowel disease, cancer of the lymphatic system, and colorectal cancer [87,88,89,90,91].

## 4. Viremia Kinetics in Non-Human Primates

Viremia is a critical parameter in assessing vaccine efficacy and predicting disease severity. In human DENV infection, high levels of viremia are usually associated with more severe disease [92,93,94,95]. In assessing vaccine efficacy, the candidate vaccine should induce an immune profile, which assures the ability to prevent or significantly reduce viremia levels [96]. Thus, it is critical for the animal to consistently develop viremia following virus inoculation. Viremia levels and kinetics in cynomolgus macaques, marmosets, and tamarins following inoculation of clinically isolated DENV1 02-17 (GenBank accession no. AB111075), DENV2 DHF0663 (GenBank accession no. AB189122), and DENV3 DSS1403 (GenBank accession no. AB189125) are shown in Table 2. DENV1 02-17 was isolated from an imported dengue fever case from Indonesia. DENV2 DHF0663 was isolated from a dengue hemorrhagic fever patient from Indonesia. DENV3 DSS1403 was isolated from an imported dengue fever case from Indonesia during the 2001 DSS epidemic. Cynomolgus macaques (CM1 to CM8) were inoculated intradermally with 4.5 × 10^6^ pfu/mL of DENV and blood samples were collected on days 0, 3, 5, 7, and 14 post-inoculation [97]. DENV genome levels were determined by Taqman real-time reverse transcriptase PCR [98]. DENV genome levels in primary infection marmosets (M1 to M8) and secondary infection marmosets (M9 to M13) were previously reported [13,21], while DENV genome levels in tamarins T3, T4, and T5 were previously reported [99]. Additionally, in marmoset models, pathological findings, including hematuria and petechiae, have been observed. Further manipulations of the model is expected to address the pathological outputs to reflect that of severe dengue in human. Cynomolgus macaques, marmosets, and tamarins consistently develop viremia following DENV inoculation. Viremia is first detected 1–3 days post-inoculation. Marmosets and tamarins consistently develop higher levels of viremia of longer duration following DENV inoculation than those of cynomolgus macaques. Cynomolgus macaques experience short-lived and low levels of viremia (average detection period: 2.6 ± 1.1 days, average peak viremia levels: 3.7 ± 2.3 log10 genome copies/mL), while marmosets and tamarins experience a longer duration of viremia (marmosets: 6.3 ± 1.9 days; tamarins: 6.4 ± 0.8 days) and higher average peak viremia levels (marmosets: 6.3 ± 1.0 log10 genome copies/mL; tamarins: 6.6 ± 0.9 log10 genome copies/mL). In primary DENV infection, cynomolgus macaques develop moderate levels of viremia, while marmosets develop high levels of viremia [13,19,21,100]. Similarly, in secondary DENV infection, cynomolgus macaques develop short-lived and low levels of viremia (average detection period: 3.0 ± 0.0 days, average peak viremia: 4.6 ± 1.9 log10 genome copies/mL), while marmosets develop higher levels of viremia and of longer duration (average detection period: 7.4 ± 2.9 days, average peak viremia: 6.8 ± 0.6 log10 genome copies/mL). Compared to another NHPs, marmosets also present with clinical signs such as fever, leucopenia, thrombocytopenia, hematuria, decreased white cell count, and are able to demonstrate some aspects of severe dengue [20,45,100,101,102]. These results suggested that marmosets are potentially useful as an animal model for studies of candidate DENV vaccines.

In human DENV infection, viremia starts 6 to 18 hours prior to the onset of illness, and average levels of peak viremia are 8.0 ± 1.0 log10 to 9.0 ± 1.0 log10 genome copies numbers/mL in cases of severe dengue [92,103,104]. *Aedes aegypti* mosquitos are thought to transfer amounts of DENV ranging from 10^3^ to 10^5^ pfu to humans for successful transmission [52,105]. Rhesus macaques develop viremia levels reaching 6.0 ± 1.0 log10 genome copies/mL when inoculated with a high dose of DENV [45]. In contrast, viremia levels reach up to 5.0 ± 1.0 log10 genome copies/mL in marmosets when inoculated subcutaneously with low dose of DENV (10^3^ pfu) in primary infection and viremia levels of 7.0 ± 1.0 log10 genome copies/mL have been detected in secondary heterologous infection [13,21]. In tamarins inoculated with 10^7^ pfu of DENV, viremia levels have reached 6.0 ± 1.0 log10 to 7.0 ± 1.0 log10 genome copies/mL [99]. In an evaluation study of one candidate vaccine, cynomolgus macaques developed low to undetectable levels of viremia following administration of the candidate vaccine [100,106]. In contrast, non-immunized marmosets develop high levels of viremia following a challenge with wild isolates, but low or undetectable levels of following administration of a candidate vaccine and a challenge with wild isolates [12]. The results of these studies indicate that marmosets could serve as an NHP model that demonstrates the protective capacity of candidate dengue vaccines.

## 5. Antibody Responses in Non-Human Primates

**Figure 1:**
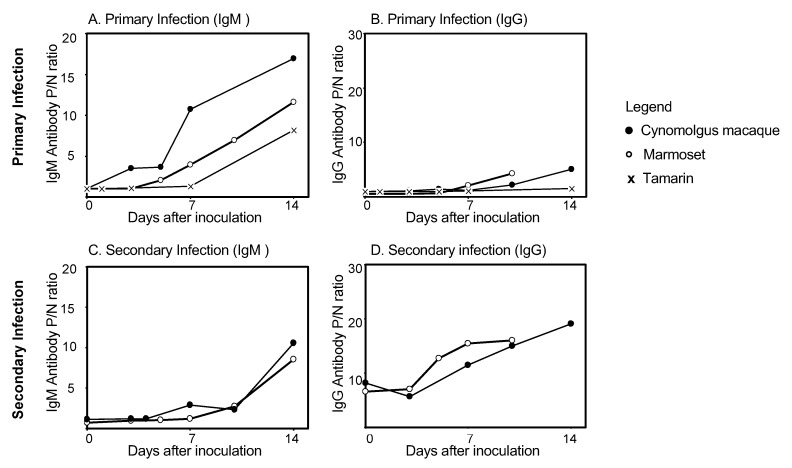
Levels of dengue virus specific IgM and IgG antibody in cynomolgus macaques, common marmosets, and tamarins: (**A**) Levels of DENV-specific IgM antibody during primary infection; (**B**) Levels of DENV-specific IgG antibody during primary infection; (**C**) Levels of DENV-specific IgM antibody during secondary infection; (**D**) levels of DENV-specific IgG antibody during secondary infection. Levels of antibody in cynomolgus macaques (○), common marmosets (●), and tamarins (**×**) were determined from day 0 to 14 after primary and secondary DENV infection. The P/N ratio indicates the positive: negative ratio. The P/N ratio was calculated by using the formula: absorbance of the test sample/absorbance of the negative control. The P/N ratio_day0_ is defined as the level of DENV-specific IgM or IgG antibody on the day of virus inoculation. DENV, dengue virus; Ig G, immunoglobulin G; Ig M, immunoglobulin M.

The presence of DENV-specific IgM and IgG antibody is used to diagnose DENV infection and to differentiate between primary and secondary infection. In human DENV infection, IgM antibodies are detected earlier than IgG antibodies in primary infection, while in secondary infection, IgG antibodies are detected earlier than IgM antibodies. The presence of DENV-specific IgM and IgG antibodies in cynomolgus macaques, marmosets, and tamarins is shown in Figure 1. Levels of DENV-specific IgM and IgG antibodies were determined by Dengue Fever IgM Capture ELISA (Focus) and Dengue IgG Indirect ELISA (PanBio), respectively [13,21,97,99,107]. Cynomolgus macaques exhibit DENV-specific IgM antibodies by day 10 after primary inoculation (mean: 6.4 ± 1.8 days), whereas marmosets demonstrate an increase in antibodies by day 7 (mean: 4.1 ± 1.5 days), and tamarins demonstrate an increase by day 14 (mean: 11.8 ± 4.5 days) (Figure 1A and 1B). By day 10, all macaques and marmosets exhibited DENV IgG antibodies. In secondary DENV infection, cynomolgus macaques exhibit a delayed DENV IgM antibody response: antibodies are detected from day 10 after inoculation, and marmosets first exhibit IgM antibodies from Day 7 after inoculation. Increases in IgM antibody levels during secondary infection occur comparatively later than in primary infection, in both cynomolgus macaques and marmosets (Figure 1C). In secondary infection, IgG antibody levels are high in both macaques (P/N ratio_day0_: 6.6) and marmosets (P/N ratio_day0_: 8.2) prior to DENV inoculation. IgG levels rise rapidly in both macaques and marmosets from day 3 after DENV inoculation (Figure 1D). Thus, in secondary infection in macaques and marmosets, as in human DENV infection, IgG levels rise rapidly, while IgM levels are significantly lower than those measured in primary infection during the early stage of secondary infection. These results indicate that antibody kinetics in these NHP models reflect those of human antibody responses during primary and secondary DENV infections.

The determination of neutralizing antibody levels is important to define the immune responses induced after vaccination in vaccine evaluation studies [108]. Neutralizing antibodies play a central role in protection against DENV infection. In addition, neutralizing antibody titers serve as an indicator of the vaccine efficacy and are a proxy measure of protection. Although high levels of neutralizing antibody correlate with protection from DENV disease, cross-reactive antibodies convey protection against heterologous DENV serotypes and are involved in infection-enhancement activities. Disease enhancement occurs when the antibody binds to DENV below the neutralization threshold. Thus, universal antibody titer cut-offs that correlate with the neutralizing antibody titers that are needed for protective immunity are needed to improve the prediction of vaccine efficacy [108].

In human DENV infection, neutralizing antibodies induced during DENV infection provide life-long protection against the homologous serotype but protection against heterologous serotype is short-lived, and cross-protection wanes over time. While cynomolgus macaques develop neutralizing antibodies against homologous serotypes within 4 weeks after primary infection, marmosets develop neutralizing antibodies against homologous serotypes by day 14 following virus inoculation in primary infection [19,21,106,109]. In human DENV infection, neutralizing antibodies against the homologous serotype remain detectable for 60 years after DENV infection [110]. Marmosets demonstrate cross-reactive neutralizing antibody against heterologous serotype following secondary heterotypic infection [13]. In addition, rapid increased in neutralizing antibody titers in marmosets following secondary heterologous infection mimics the antibody response pattern in human DENV infection [12,13,109].

## 6. Conclusions and Future Directions

The global burden of dengue continues to increase annually and the lack of a widely available dengue vaccine contributes to this trend. Unlike influenza, enteric bacteria, and malaria, there is currently no licensed specific therapy for dengue; thus, human challenge models cannot be used, and animal models remain an important component in dengue vaccine evaluation studies. However, the development of dengue vaccines has been hampered by the lack of a reliable animal model for dengue vaccine efficacy studies. Although there are several animal models for dengue, there remains a need for a robust animal model that faithfully reflects the immune response patterns and clinical signs that is comparable to that of human DENV infection, including the age-associated clinical signs, and sustains viral replication. While mouse models are widely used to study the disease pathogenesis, these models are less sensitive to clinical DENV strains, and so the dengue antibody response may not accurately reflect that of human DENV infection. The NHP model is a potential useful animal model for disease pathogenesis because NHPs are hypothesized to be hosts of DENV in sylvatic cycles and the close genetic proximity between NHP and human. Cynomolgus macaques experienced short-lived and low levels of viremia and exhibited an antibody response pattern similar to those of human DENV infection but the high cost of maintenance and the lack of clinical manifestations limit the use of cynomolgus macaques as an animal model for dengue. In contrast, marmosets consistently develop high levels of viremia, and have exhibited antibody response patterns and clinical signs similar to those of human DENV infection, and the cost of maintenance of marmosets are generally lower than that of other NHPs. Thus, the marmoset model is a potentially useful animal model in DENV challenge studies and vaccine development. Studies on cellular immunity, cytokine profile and the protective levels of neutralizing antibodies against clinically isolated DENV using marmoset as an animal model will further determine the suitability of marmoset as an animal model for DENV.

## Figures and Tables

**Table 1:** Summary of the benefits and limitations of animal models of dengue infection.

Type of Animal Model	Benefits of Use This Model	Limitations	References
Immunocompetent mice (C57BL/6 mice, BALB/c mice)	Provide insight on the mechanistic contribution of host immune response to immunopathogenesis	Limited data on infection by natural route of infection (i.e., mosquito bite) Less sensitive to challenge with clinical DENV strains Low/ undetectable systemic infective viremia Lack of clinical manifestations	[22,23,24,25]
Interferon alpha/beta/gamma receptor knock-out mice) (AG129 mice)	Help to understand the disease pathogenesis due to the ability to demonstrate disease pathology and viral replication following DENV injection Provide insight on the efficacy of the vaccine due to the ability to demonstrate protective effects and induction of neutralizing antibody in DENV vaccine candidate study	Immune system response may not faithfully reflect those of natural hosts Limited utility in studies on interaction between humoral and cell-mediated immune response in vivo. Lack of clinical manifestations Severe disease development is age-dependent, thus the animal’s limited lifespan is a limitation	[26,27,28,29,30]
IFN -/- mice (IFNAR^-/-^ mice)	Allows the investigation of T-cell responses relevant to DENV vaccine design and better modeling the T-cell response during DENV infection	Limited capability in mounting full immune response due to the lack of IFN-αβ and γ receptor Lack of clinical manifestations	[31,32,33]
Humanized mice (hu-NSG mice, NOD/SCID mice, NOD-*scidIL2Rγ^null^* mice, RAG2^-/-^^γ^_c_^-/-^mice, BLT-NOD/SCID mice)	Allows the investigation of antibody response and cytokines following DENV infection Ideal to study disease pathogenesis due to the presence of clinical manifestation and viremia	The viremic period is not consistent with human DENV infection Requires highly technical process including the cells used for engraftment and consistently high levels of engraftment	[34,35,36,37,38,39,40,41]
Non-human primates (rhesus macaque, bonnet monkey, olive baboons, African green monkey)	Natural hosts in sylvatic DENV cycle Allows the investigation of immune response because it has been shown to be similar to human DENV infection Provides insights on correlation between protection and disease pathogenesis Demonstrates measurable viremia Useful to measure protection conferred by vaccination or passively acquired antibody	High cost of maintenance Lack of clinical manifestations	[42,43,44,45,46,47,48,49,50]
Dengue human infection model (DHIM)	Most biologically relevant model Provides relevant insights into immune response to dengue virus Cost-effective avenue for testing drug efficacy before large scale clinical trials	Limited accessibility, ethical issues and regulatory restrictions No licensed specific therapy for dengue virus, potential risk for severe disease development Long-term risk of severe dengue development in participants after study (natural infection) End-point is potentially unethical or difficult to measure Variances in immunology and genetic background	[51,52,53,54,55,56,57]

**Table 2:** Comparison of dengue viral RNA levels (log10 genome copies/mL) in plasma of cynomolgus macaques, common marmosets, and tamarins during primary and secondary dengue virus infection.

Type of Infection	Animal ID	Inoculated Virus	Dengue Viral RNA Copy Numbers (log10 Genome copies/mL)								
			Days after Inoculation								
			0	1	2	3	4	5	7	10	14
**Primary**	**(A) Cynomolgus macaques**										
	*Group 1*										
	CM1	DENV1 01-27	NT	NT	NT	2.8	NT	-	-	-	-
	CM2		NT	NT	NT	-	NT	-	-	-	-
										
	*Group 2*										
	CM3	DENV2 DHF0663	NT	NT	NT	6.5	NT	-	-	-	-
	CM4		NT	NT	NT	3.1	NT	-	-	-	-
	CM5		NT	NT	NT	4.1	NT	-	-	-	-
	CM6		NT	NT	NT	7.2	NT	-	-	-	-
										
	*Group 3*										
	CM7	DENV3 DSS1403	NT	NT	NT	2.5	NT	-	-	-	-
	CM8		NT	NT	NT	3.6	NT	-	-	-	-
										
	**(B) Marmosets ^1^**										
	*Group 4*										
	M1	DENV1 02-17	-	NT	NT	5.6	NT	5.7	-	-	-
	M2		-	NT	7.0	NT	NT	6.5	7.7	6.0	-
										
	*Group 5*										
	M3	DENV2 DHF0663	-	NT	NT	7.2	NT	5.0	-	NT	-
	M4		-	NT	NT	7.5	NT	6.8	5.4	NT	-
	M5		-	NT	4.5	NT	6.0	NT	4.0	NT	-
	M6		-	NT	5.0	NT	6.3	NT	4.2	NT	-
										
	*Group 6*										
	M7	DENV3 DSS1403	-	NT	NT	-	NT	4.7	-	-	-
	M8		-	NT	4.9	NT	5.6	NT	-	NT	-
										
	**(C) Tamarins ^1^**										
	*Group 7*										
	T1	DENV2 DHF0663	-	6.4	NT	6.1	NT	4.2	-	NT	NT
	T2		-	7.3	NT	7.5	NT	6.3	4.2	NT	NT
										
	*Group 8*										
	T3	DENV2 DHF0663	-	5.3	NT	6.2	NT	NT	4.5	-	-
	T4		-	4.7	NT	4.6	NT	NT	5.4	-	-
	T5		-	5.3	NT	6.3	NT	NT	6.2	-	-
										
**Secondary**	**(D) Cynomolgus macaques**										
	*Group 9*										
	CM1	DENV2 DHF0663	NT	NT	NT	6.2	NT	-	-	-	-
	CM2		NT	NT	NT	6.2	NT	-	-	-	-
										
	*Group 10*										
	CM3	DENV3 DSS1403	NT	NT	NT	3.0	NT	-	-	-	-
	CM4		NT	NT	NT	2.8	NT	-	-	-	-
										
	**(E) Marmoset ^2^**										
	*Group 11*										
	M9	DENV2 DHF0663	-	-	NT	6.7	NT	NT	4.5	NT	-
	M10		-	-	NT	6.2	NT	NT	-	NT	-
	M11		-	-	NT	6.4	NT	NT	3.9	NT	-
										
	*Group 12*										
	M12	DENV3 DSS1403	-	-	7.0	NT	NT	6.5	5.2	4.7	-
	M13		-	-	7.5	NT	NT	7.7	6.0	4.2	-

(-) indicates below detection levels; NT indicates not tested or samples were not collected. ^1^ DENV genome levels in marmosets M1 to M8 and tamarins T3, T4, and T5 were previously reported [12,13]. ^2^ DENV genome levels in marmosets M9 to M13 were previously reported [20,21]. Cynomologous macaques (CM) were infected as previously reported [97,98].
